# Contact allergy to fragrances: current clinical and regulatory trends 

**DOI:** 10.5414/ALX01604E

**Published:** 2017-08-04

**Authors:** W. Uter

**Affiliations:** Institut für Medizininformatik, Biometrie und Epidemiologie, Universität Erlangen-Nürnberg, Erlangen, Germany

**Keywords:** contact allergy, fragrances, consumer products

## Abstract

Abstract. Several fragrances are important contact allergens. Compared to the immense multitude of more than 2,500 fragrances used in cosmetics, the spectrum of single substances and natural extracts used for patch testing appears limited, albeit comprising the supposedly most important contact allergens. The present review summarizes the most important results of the opinion of the Scientific Committee on Consumer Safety on fragrance allergens in cosmetic products from July 2012. Clinical results beyond abovementioned screening allergens, animal results in terms of the LLNA and structure activity considerations point to 100 single substances and extracts, respectively, which, in addition to those 26 already identified, must be considered contact allergens, and the presence of which should be declared in cosmetics. In case of the most commonly used fragrance terpenes limonene and linalool hydroperoxides resulting from autoxidation constitute the major allergens. These have become available as patch test material recently. Altogether 12 single substances have caused a (very) high number of published cases of sensitization. Thus their use concentration should be (further) reduced or, in case of hydroxyisohexyl 3-cyclohexene carboxaldehyde (HICC, e.g., Lyral^®^), use should be abandoned altogether. This is also recommended in case of oak moss and tree moss due to their content of the strong sensitizers atranol and chloroatranol. As generic maximum dose for the remaining 11 single substances 0.8 µg/cm^2^ are suggested, which corresponds, under conservative assumptions, a maximum concentration of 100 ppm in the finished product.

German version published in Allergologie, Vol. 36, No. 12/2013, pp. 550-560

Abbreviations HICC: hydroxyisohexyl 3-cyclohexene carboxaldehyde; IFRA: International Fragrance Association (www.­ifraorg.org); INCI: International Nomenclature of Cosmetic Ingredients (siehe http://ec.­europa.eu/consumers/cosmetics/cosing/); IVDK: Informationsverbund Dermatologischer Kliniken (Information Network of Departments of Dermatology) (www.ivdk.org); LLNA: local lymph node assay; (Q)SAR: (quantitative) structure activity relationship; REACH: registration, evaluation, authorization and restriction of Chemical substances; SCCS: Scientific Committee on Consumer Safety; URL: Uniform Resource Locator (internet address).

This review paper summarizes the current knowledge of contact allergies to fragrances. It is mainly based on the opinion of the Scientific Committee on Consumer Safety (SCCS) published in July 2012 (http://ec.europa.eu/health/scientific_committees/consumer_safety/docs/sccs_o_102.pdf; last accessed May 13, 2013; [1]). While the clinical and allergological basics are assumed to be known to the reader, the clinical epidemiology of the most common fragrance contact allergens are presented in a more detailed way than in [2]. Furthermore, experimental data (LLNA) and knowledge on the (bio-)activation of substances and haptens as well as chemical considerations of structure-effect relationships are used to identify fragrances that pose a particular problem and make step-by-step preventive measures necessary. To keep the list of references concise, only selected, exemplary references were included; for further information and a complete list of references please refer to [1] and to the above-mentioned opinion of the SCCS, which is available as an open-access publication on the above-mentioned website. The review paper presented here does not cover substances or extracts that are banned from use in cosmetic products (Annex II of the Cosmetics Regulation) [3]. 

## Allergens for screening 

A mixture of fragrances, as is used in a perfume or as a perfume component of a cosmetic product, contains several to several hundred single fragrances. The CosIng database (http://ec.europa.eu/consumers/cosmetics/cosing/; last accessed May 13, 2013) includes more than 2,500 substances or extracts categorized as a fragrance. Of this enormous multitude, several mixtures consisting of what have been defined to be the most common fragrance allergens, plus (since about 10 years) one single synthetic fragrance, are currently used as the patch test standard series for clinical diagnosis. 

For several decades, fragrance mix I, a mixture of 1% each of the 7 synthetic substances (INCI nomenclature) amyl cinnamal, cinnamyl alcohol, cinnamal, eugenol, geraniol, hydroxycitronellal, isoeugenol, and oak moss (Evernia prunastri) in petrolatum, together with 5% sorbitan sesquioleate, as an emulsifier, has been used. In Europe, the prevalence of sensitization in consecutively-tested patients lies between 4.5% and 14.8%; worldwide, the difference is even larger. In central Europe, the frequency was 7.3% for the years 2005 – 2008, according to data collected by the Information Network of Departments of Dermatology (IVDK) [4]. The prevalence of sensitization in the general population lies between 1% and 3%, according to most studies. 

Fragrance mix II was introduced in 2005 [5]. It contains 6 synthetic fragrances in concentrations between 0.5% and 5%: citronellol (0.5%), citral (1%), coumarin (2.5%), hydroxyisohexyl 3-cyclohexene carboxaldehyde (HICC, 2.5%), farnesol (2.5%), and α-hexyl-cinnamal (5%); total concentration 14% in petrolatum. In European studies, the prevalence of sensitization is 0.6 – 4.9% in consecutively-tested patients and thus is markedly lower than that of fragrance mix I. However, this additional test is able to identify a significant additional proportion of fragrance-sensitized patients [6]. 

The most important ingredient in fragrance mix II is hydroxyisohexyl 3-cyclohexene carboxaldehyde (HICC, also known as Lyral^®^), which is additionally tested in 5% (pet.) in the standard series due to its significance. Around the year 2000, high concentrations of HICC were used in cosmetic products, e.g., in deodorants. This led to a downright epidemic of HICC sensitizations, which still has not been sufficiently controlled by self-regulatory measures applied by the industry (“IRFA standards”). In central Europe (according to IVDK data), the prevalence of HICC sensitization was almost 20% in 2011 [7]; in Denmark, it was 2.5% [8]. Interestingly, there are important differences among European countries, with lower prevalence in the south [9]; in the USA, sensitization to HICC is also significantly less frequent [10], which suggests marked differences regarding exposure (use in products, consumer habits). 

Another mixture that has been used as a screening allergen for years is Balsam of Peru (Myroxylon pereirae, INCI). While the balsam as such is not used in cosmetic products in Europe, extracts and distillates are [11]. Furthermore, exposure through topic drugs has to be considered in some regions. With a prevalence of sensitization between 3.9% and 8.0% in consecutively-tested patients in Europe and strong associations with other fragrance allergens, Balsam of Peru is a “traditional” but still common allergen, although the composition and the role of individual ingredients as sensitizing agents has not yet been fully explained. Turpentine, as an allergen, is significantly less common; currently, the prevalence of sensitization in consecutively-tested patients is usually no higher than 2%. The content of relevant substances varies widely, according to their origin; nevertheless, turpentine is a common raw material in the perfume industry and contains substances (terpenes) that come from other sources. 

## Activation of substances to sensitizers: pre- and prohaptens 

To our current knowledge, most fragrances are haptens, which, after binding to proteins, become allergens and are able to induce an immune reaction (sensitization and subsequent elicitation). Some fragrances need to be activated before they can bind to proteins. If this activation takes place outside the body, for example by autoxidation or photoactivation, the substance is a prehapten. Prohaptens, on the other hand, are transformed into immunogenic haptens within the skin, usually by enzyme catalysis. It is not always clear whether a substance is a prehapten, a prohapten, or both, as both activation pathways can result in the same products, such as geraniol (geranial, epoxy-geraniol, and epoxy-geranial), for example [12, 13]. 

From an allergological point of view, the most common reaction products of prehaptens are hydroperoxides, but also secondary-reaction products like aldehydes and epoxides can contribute to sensitizing potential [14]. In animal experiments, the oxidation products of terpenes, like limonene, linalool, geraniol, and linalyl acetate, which are frequently used as fragrances, have been identified to be markedly more potential allergens than the nonoxidized raw substances. These results concur with clinical trials in which patch tests using oxidized terpenes resulted in a significantly higher prevalence of sensitization than patch tests using nonoxidized material. Interestingly, the oxidation of different substances results in identical, or at least similar, reaction products, which could explain cross-reactivity. As oxidation can be avoided or at least delayed by the addition of antioxidant agents, these are used more and more frequently. However, it has to be closely monitored as to whether the antioxidant agents, like the frequently-used butylated hydroxytoluene, can themselves cause allergies. 

Various enzyme systems in the skin are able to metabolize foreign substances (xenobiotics), including prohaptens. The aim is “detoxification”; what happens, however, is the transiently increased harmfulness of a substance in terms of a sensitizing effect. The influence on allergenicity has only been investigated in relatively few substances so far, e.g., in α-terpenes, geraniol, cinnamyl alcohol, eugenol, and isoeugenol. Predictive in-vitro tests, which will gain importance once animal experiments on ingredients of cosmetic products expire, have so far not included this aspect. In clinical practice, i.e., for patients, the process of bioactivation is of high importance as it leads to the necessity to take into account the exposure to mother substances that produce the reaction product against which sensitization is present (e.g., isoeugenol acetate results in isoeugenol after scission of the ester bond, and cinnamyl alcohol is metabolized to cinnamal) [15, 16]. 

## Clinical results 

The SCCS’s opinion followed a structured approach in its evaluation of whether and to what extent a fragrant substance or mixture has to be regarded as allergically relevant [1]. The first step was to sift through the publications on clinical cases of sensitization. When at least two independent centers reported either well-documented case reports or several positive patch test results in a series of patients, the substance or extract was categorized as “established allergen in humans”. The results are presented in Table 1 and Table 2. Only if no clear classification could be obtained based on human data, which – if sufficiently validated – is always preferred to other data, results from animal experiments and structural chemistry were additionally taken into account (see below). 

## Experimental data derived from the local lymph node assay (LLNA) 

To identify further potential allergens, the SCCS also collected data from animal experiments. Some of these data were provided by the industry [17], others were taken from two published review papers [17]. All data were derived from the LLNA; in addition, there will very probably be further, unpublished LLNA results, further trials as well as data derived from other methods, like the “guinea pig maximization test” (GPMT) or the Buehler test. An EC3 value (i.e., triplication of the stimulation index) was present in 55 of the 70 investigated fragrances, with this value being > 2% in 50 of these substances. According to the traditional classification, fragrances are thus mainly categorized as “moderate” or “weak” allergens. Despite this, fragrances are among the most frequent allergens; this must be due to certain characteristics of exposure, like repeated use, mixture of numerous substances [20], or use in problematic areas (e.g., axilla or hands with lesioned skin). Furthermore, the real allergenic potential will probably be underestimated in some cases, as illustrated by the problematic allergen HICC (see above); its EC3 value was 17.1% in the LLNA; in comparison, the value of benzyl benzoate was 17%, and, based on human data, this substance indeed has to be seen as a weak allergen. Table 3 shows LLNA results for substances/mixtures that have not yet been identified as “established allergens in humans” (see above). 

## Structure activity relationship (SAR) 

The ability of a substance to act as a hapten, be it after (bio-)activation, significantly depends on its bonding capacity to skin proteins. This characteristic can frequently be deduced from the chemical structure of the molecules when “structural alerts” are observed [21]. A further option is to study the quantitative structure activity relationships (QSAR); this investigation is based on experimental findings on reactivity and other substance-specific data. However, for many fragrances, no quantitative data are available. Furthermore, the sometimes decisive (bio-)activation [14] makes valid modeling difficult. Therefore, fragrances that are important in terms of exposure, but for which insufficient human or experimental data were present, were categorized for the SCCS opinion based on the consenting expert opinion of the involved chemists. Table 4 shows the fragrances for which a sensitizing effect is predicted (“++”) or possible (“+”) and for which additionally (i) human data are present that alone were not sufficient to categorize a substance as “established allergen in humans” or (ii) findings from animal experiments suggest an important sensitizing potency. The latter was not demonstrated by the above-mentioned, separately-considered experimental studies but rather on the basis of an “R43”-label according to REACH. 

## Exposure 

Skin contact with fragrances can be present through the personal use of cosmetic or household products etc., but it can also take place when using pharmaceutical products or occupational substances, having close contact with other people, and even over the air. In addition to a substance’s intrinsic allergenic potency, the following exposure factors are important for the risk of sensitization or elicitation: area dose (usually presented as µg/cm2), vehicle effects, simultaneous presence of irritants or further potential allergens, time and frequency of exposure, localization, skin status, and occlusion (e.g., in skinfolds, under clothing or personal protective equipment). In a series of tests, either the qualitative formulations (INCI declaration, e.g., [22, 23]) or – by chemical analysis – the quantitative compositions [24, 25] were studied with regard to relevant fragrances. The most frequently-identified substances were – with certain differences between the types of products – limonene and linalool. The relatively limited quantitative data show that the content of the most common allergens in perfumes and deodorants has markedly decreased [24]. However, it was also found that the mean concentration of atranol, one of the most common allergens in oak moss and tree moss, rather increased from 2004 to 2007, while the chloroatranol concentration decreased [25]. 

Some fragrances can, for example, be used as repellants, insecticides, or bactericides (see, e.g., biocide directive 98/8/EC). The use of benzyl benzoate as a scabicide, farnesol as a bacteria-inhibiting additive in deodorants, or benzyl alcohol as an antioxidant in external agents are only three of the better-known examples. This leads to additional manners of exposure to these fragrances beyond their use in cosmetic products and also beyond their usual function as a fragrance. The same holds true for the use of certain fragrances or natural extracts in aromatherapy, massage oils, or the like. 

With regard to exposure from various sources, it has to be taken into account that particularly the hands are exposed not only to fragrances but also to other allergens when applying body lotions, facial creams, or other products. This is called “aggregate exposure”; by cumulative effects, critical area doses can be exceeded, thus facilitating sensitization or elicitation. 

## Dose-effect relationships and thresholds 

In general, risk estimation is based on data on hazard (i.e., sensitization potency), exposure, and dose-effect relationship at induction. For ethical reasons, human induction studies are objectionable today, and the industry only uses them to verify an elsewhere-deduced “no effect level” (NOEL), therefore, usually no cases of sensitization are observed; but it also has to be taken into account that the samples sizes are always very small. Thus, only data on elicitation (i.e., studies in sensitized patients) are available to evaluate dose-effect relationships. Ideally, these kinds of studies would be (i) available for all relevant (i.e., problematic) fragrances, (ii) performed as repetitive open application test (ROAT) according to the standardized guidelines for cosmetic application [26], and (iii) carried out for various types of products. An area dose that does not lead to an allergic reaction in most sensitized patients (e.g., an “eliciting dose (ED)10”, which is tolerated by 90% of patients) can usually be regarded as safe with regard to the primary prevention of an induction. 

However, a ROAT study design is highly complex so that triggering thresholds are available for only few fragrances:

- Isoeugenol at a concentration of 63 ppm in deodorants leads to an allergic reaction in 3/13 sensitized patients. In a ROAT study that used ethanol as a vehicle (representing “hydroalcohol” perfume bases), 2.2 µg/cm^2^ triggered a reaction in 42% in one investigation, and 5.6 µg/cm^2^ triggered a reaction in 63% of isoeugenol-sensitized patients in another investigation.
- Cinnamal 320 ppm in deodorant triggered an allergic contact eczema in 2/8 sensitized patients, 100 ppm triggered the same reaction in 1/9 sensitized patients, and a ROAT using 0.1% in ethanol triggered allergic contact eczema in 44% of sensitized patients.
- In a ROAT, hydroxycitronellal 320 ppm in deodorant led to a positive reaction in 4/7 sensitized patients.
- HICC 200 ppm in deodorant triggered allergic reactions in 9/14 sensitized patients. An ED10 of 1.2 µg/cm^2^ for ethanol and an ED10 of 4.9 µg/cm^2^ for a cream base were detected in a larger ROAT study carried out by the IVDK [23]. This corresponds to concentrations of 270 ppm (alcohol base) and 88 ppm (cream). In a further ROAT study, 15.3 µg/cm^2^ in ethanol led to a positive reaction in 61% of patients; using an ethanol/water mixture, the ED10 was found to be 0.064 µg/cm^2^ in another investigation.

Chloroatranol, the allergologically relevant component of *Evernia prunastri* (oak moss), led to an allergic reaction in 92% of oak moss-sensitized patients at particularly low area doses of 0.025 µg/cm^2^. In ROAT, even extracts, in which the atranol and chloroatranol contents could be reduced to 75 ppm (3.4%) and 25 ppm (1.8%), respectively, triggered allergic reactions in most patients with a sensitization against oak moss [27] so that sufficient reduction of allergens does not seem to be achievable by this means. 

As the data are incomplete and cannot be applied to each fragrance, the SCCS opinion suggests using a generic threshold of 0.8 µg/cm^2^. This value is based on the observation that this area dose can be regarded as the mean ED10 in several other allergens, including metals and biocides. Because a certain area dose corresponds to different concentrations in different products (depending on the base, frequency of use, etc. [26]), the suggested threshold value of 0.8 µg/cm^2^ was translated to a maximum concentration of 100 ppm (0.01%) based on the most critical base, i.e., deodorants. 

## Prevention 

In fragrance contact allergy, as in general, a distinction between primary and secondary prevention is possible. While primary prevention aims to avoid sensitization from the beginning, secondary prevention tries to avoid relapses, i.e., episodes of allergic contact eczema, in sensitized patients. 

For **primary prevention**, there are various measures, and some of them are even carried out before the market launch of a fragrance: Substances that turn out to be (too) sensitizing are excluded from use in cosmetic products (see CosIng, entries in Annex II of the Cosmetics Directive). Unfortunately, these screening mechanisms are not perfect so that many fragrances with a known sensitizing effect are present in cosmetics and other consumer products (see above). Thus, it is necessary to monitor contact allergies in post-marketing surveillance programs in order to detect problematic substances and to carry out the necessary interventions. The latter are primarily the limitation of the maximum-allowed concentration applied and, if this measure is not sufficiently effective, the ban of the substance in question. In an effort towards self-regulation, the industry, through its research institute IFRA (www.ifraorg.org), has developed numerous standards for problematic substances. However, these standards are nonbinding, cover most but not all companies, and do not always adhere to clinicoepidemiological findings with sufficient consistency and timeliness. Therefore, the SCCS opinion found it necessary to limit the concentration of 12 individual substances, which were considered to be particularly problematic (Table 1, bold print), to the above-mentioned generic maximum concentration. For natural extracts, a limitation of the concentration did not seem feasible because of lack of data and varying composition; exceptions are the 12 above-mentioned problematic ingredients, even if they are used in extracts, when their concentration in the final product exceeds the proposed threshold value. It has been recommended to not use HICC and atranol-/chloroatranol-containing extracts from *Evernia spp.* in cosmetic products because previous efforts to limit the concentration were not sufficiently effective. 

Successful **secondary prevention** is based on adequate diagnostic work-up. Only if the substances suspected to have caused the allergic contact eczema are (i) identified and (ii) tested on the skin, exposure to these agents can be avoided in the future. Thus, secondary prevention is based on information on ingredients – in this case, mainly cosmetic products, but in general these can also be, e.g., occupational substances. With regard to cosmetic products, the introduction of the INCI declaration has led to a significant progress – as long as allergists use exactly the INCI terminology to inform the patient, e.g., using an “allergy pass”. When the INCI declaration was introduced, the individual fragrances (if used as a perfume and not as an antioxidant agent, like benzyl alcohol, or as an antimicrobial additive, like farnesol) were not included and globally denoted “perfume”. The first step to limit this privilege of “non-information” was the introduction of the requirement for labeling of 24 fragrances and 2 extracts [29]. The current SCCS opinion has identified 71 further individual substances and 29 further extracts that (i) are “established allergens in humans” (Table 1, Table 2), (ii) are shown to be sensitizing in the LLNA (Table 3), or (iii) have a high probability to be sensitizing agents (Table 4). For this reason, the requirement for labeling should be extended to 127 substances or extracts. How exactly this could be done, apart from or in addition to the current labeling policy on product packages, remains to be discussed. Furthermore, allergists and manufacturers of patch tests are facing the challenge of having to develop a relatively high number of new formulations to further optimize diagnostic work-up. Some extracts have already been made available, and three of them have been used by the German contact allergy group or the IVDK within the standard series; it was found that the tested allergens were frequent allergens [30]. An optimized diagnostic work-up is possible – at least theoretically –, if both the requirement for labeling and the range of available patch test substances includes further important fragrance allergens. Whether this level of diagnostic work-up can be made available in each dermatology practice or only in more specialized institutions, also at the currently-reached stage (26 “Annex fragrances”), remains to be discussed elsewhere. 

## Acknowledgment 

I wish to thank my colleagues A. Börje (Gothenburg), C. Chambers (Dublin), J.D. Johansen (Copenhagen), A.-T. Karlberg (Gothenburg), C. Lidén (Stockholm), S.C. Rastogi (Copenhagen), D.W. Roberts (Liverpool), I.R. White (London, President), and Ms. Karin Kilian (Directorate General Consumer Protection), together with whom I was able to develop the expert opinion that constitutes the basis of this review paper for the SCCS work group “Sensitization and Fragrances” between 2009 and 2012. 

## Conflict of interest 

W.U. received remunerations and compensation for travel expenses for lectures given for the cosmetic industry. 

**Table 1.** Known contact allergens in humans: single substances. Substances with an “alarming” prevalence of sensitization (100 – 1,000 reported cases: +++; > 1,000 reported cases: ++++) are presented in bold. ox. = oxidized; ­n.-ox. = nonoxidized; r.t. = rarely tested.

INCI name (or, if no INCI name exists, usual name according to CosIng)	CAS number	Number of cases (see text)
Acetylcedrene	32388-55-9	+
Amyl cinnamal	122-40-7	++
Amyl cinnamyl alcohol	101-85-9	++
Amyl salicylate	2050-08-0	+
trans-anethole	4180-23-8	+ (r.t.)
Anise alcohol	105-13-5	+
Benzaldehyde	100-52-7	+
Benzyl alcohol	100-51-6	++
Benzyl benzoate	120-51-4	++
Benzyl cinnamate	103-41-3	++
Benzyl salicylate	118-58-1	++
Butylphenyl methylpropional (e.g., Lilial ^®^)	80-54-6	++
Camphor	76-22-2/464-49-3	+ (r.t.)
beta-Caryophyllene	87-44-5	n.-ox.: + ox.: +
Carvone	99-49-0/6485-40-1/2244-16-8	+ (r.t.)
**Cinnamal**	**104-55-2**	**+++**
**Cinnamyl alcohol**	**104-54-1**	**+++**
**Citral**	**5392-40-5**	**+++**
Citronellol	106-22-9/1117-61-9/7540-51-4	++
**Coumarin**	**91-64-5**	**+++**
Rose Ketone-4 (Damascenone)	23696-85-7	+ (r.t.)
alpha-Damascone (TMCHB)	43052-87-5/23726-94-5	++
cis-beta-Damascone	23726-92-3	+
delta-Damascone	57378-68-4	+
Dimethylbenzyl carbinyl acetate (DMBCA)	151-05-3	+
**Eugenol**	**97-53-0**	**+++**
**Farnesol**	**4602-84-0**	**+++**
**Geraniol**	**106-24-1**	**+++**
Hexadecanolactone	109-29-5	+ (r.t.)
Hexamethylindanopyran	1222-05-5	++
**Hexyl cinnamal**	**101-86-0**	**++**
**Hyydroxyisohexyl 3-cyclohexene carboxaldehyde (HICC)**	**31906-04-4/51414-25-6**	**++++**
**Hydroxycitronellal**	**107-75-5**	**+++**
**Isoeugenol**	**97-54-1**	**+++**
**alpha-Isomethyl ionone**	**127-51-5**	**++**
(DL)-Limonene	138-86-3	++ (n.-ox.) +++ (ox.)
Linalool	78-70-6	++ (n.-ox.) +++ (ox.)
Linalyl acetate	115-95-7	+ (n.-ox.) ++ (ox.)
Menthol	1490-04-6/89-78-1/2216-51-5	++
6-Methyl coumarin	92-48-8	++
Methyl 2-octynoate	111-12-6	++
Methyl salicylate	119-36-8	+
3-Methyl-5-(2,2,3-trimethyl-3-cyclopentenyl)pent-4-en-2-ol	67801-20-1	++ (r.t.)
alpha-Pinene and beta-Pinene	80-56-8 and 127-91-3, resp.	++
Propylidene phthalide	17369-59-4	+ (r.t.)
Salicylaldehyde	90-02-8	++
alpha-Santalol and beta-Santalol	115-71-9 and 77-42-9, resp.	++
Sclareol	515-03-7	+
Terpineol (isomere mix)	8000-41-7	+
alpha-Terpineol	10482-56-1/98-55-5	
Terpinolene	586-62-9	+
Tetramethyl acetyloctahydronaphthalenes	54464-57-2/54464-59-4/68155-66-8/68155-67-9	+
Trimethyl- benezenepropanol (Majantol)	103694-68-4	++
Vanillin	121-33-5	++

**Table 2.** Known contact allergens in humans: extracts (essential oils). Substances with an “alarming” prevalence of sensitization (100 – 1,000 reported cases: +++; > 1,000 reported cases: ++++) are presented in bold. r.t. = rarely tested.

INCI name (or, if no INCI name exists, usual name according to CosIng)	CAS number	Number of cases (see text)
***Cananga odorata*** **(ylang-ylang oil)**	**83863-30-3; 8006-81-3**	**+++**
*Cedrus atlantica* bark oil (cedar oil)	92201-55-3; 8000-27-9	++
*Cinnamonum cassia leave oil *(cassia oil) *Cinnamonum zeylanicum* bark oil (cinnamon oil)	8007-80-5 84649-98-9	++ (r.t.)
*Citrus aurantium amara* flower/peel oil (neroli oil)	8016-38-4; 72968-50-4	++
*Citrus bergamia* peel oil expressed (bergamot oil)	89957-91-5	+ (r.t.)
*Citrus limonum* peel oil expressed (lemon oil)	84929-31-7	++
*Citrus sinensis *(syn.: *aurantium dulcis*) peel oil expressed (Orange oil)	97766-30-8; 8028-48-6	++
*Cymbopogon citratus/schoenanthus* oils (lemongrass oil)	89998-14-1; 8007-02-1; 89998-16-3	++
*Eucalyptus spp.* leaf oil (eucalyptus oil)	92502-70-0; 8000-48-4	++
***Eugenia caryophyllus* leaf/flower oil (clove oil)**	**8000-34-8**	**+++**
***Evernia furfuracea* extract (tree moss)**	**90028-67-4**	**+++**
***Evernia prunastri* extract oak moss)**	**90028-68-5**	**+++**
***Jasminum grandiflorum*/officinale (jasmin abs.)**	**84776-64-7; 90045-94-6; 8022-96-6**	**+++**
*Juniperus virginiana* (cedarwood oil)	8000-27-9; 85085-41-2	++
*Laurus nobilis* (laurel oil)	8002-41-3; 8007-48-5; 84603-73-6	++
*Lavandula hybrida* (lavandula hybrida extract)	91722-69-9	+ (r.t.)
*Lavandula officinalis *(*lavandula angustifolia* herb extract)	84776-65-8	++
*Mentha piperita* (peppermint oil)	8006-90-4; 84082-70-2	++
*Mentha spicata* (spearmint oil)	84696-51-5	++
***Myroxylon pereirae* (extracts, distillates) (Balsam of Peru)**	**8007-00-9;**	**++++**
*Narcissus spp.* (Narcissus abs.)	miscellaneous	++
*Pelargonium graveolens* (geranium oil Bourbon)	90082-51-2; 8000-46-2	++
*Pinus mugo/pumila* (pinus mugo twig leaf extract)	90082-72-7/97676-05-6	++
*Pogostemon cablin *(patchouli oil)	8014-09-3; 84238-39-1	++
*Rose* flower oil (*Rosa spp.*) (rose oil)	miscellaneous	++
***Santalum album* (sandalwood oil)**	**84787-70-2; 8006-87-9**	**+++**
***Turpentine* (oil)**	**8006-64-2; 9005-90-7; 8052-14-0**	**++++**

**Table 3.** Results of the local lymph node assay (LLNA) for fragrances that have not been categorized as “established allergen in humans” (for a presentation of all substances see [1]). In ascending order of EC3 value (low values represent higher allergenic potency).

Substance (INCI name or name of perfume according to CosIng)	CAS number	EC3 value		Reference
		%	molar	
Hexyl salicylate	6259-76-3	0.18	0.008	[17, 18]
2-Hexylidene cyclopentanone	17373-89-6	2.4	0.14	[17]
Methyl octine carbonate	111-80-8	2.5	0.15	[17]
trans-2-Hexenal	6728-26-3	2.6	0.26	[17]
Phenylacetaldehyde	122-78-1	3	0.25	[17, 19]
Allyl phenoxyacetate	7493-74-5	3.1	0.16	[17]
4-tert.-Butyldihydrocinnamaldehyde	18127-01-0	4.3	0.23	[17]
Methylcinnamic aldehyde	101-39-3	4.5	0.31	[17, 19]
2-Methoxy-p-cresol (2-Methoxy-4-methylphenol)	93-51-6	5.8	0.42	[17, 19]
Dibenzyl ether	103-50-4	6.3	0.32	[17]
Isocyclocitral	1335-66-6	7.3	0.48	[17]
2,3-Dihydro-2,2,6-trimethylbenzaldehyde	116-26-7	7.5	0.50	[19]
Perillaldehyde (p-Mentha-1,8-dien-7-al)	2111-75-3	8.1	0.54	[17, 19]
3-(p-Cumenyl)-2-methylpropionaldehyde (p-Isobutyl-a-methyl hydrocinnamaldehyde)	6658-48-6	9.5	0.46	[17]
Methylundecanal	110-41-8	10	0.54	[19]
Methylenedioxyphenyl methylpropanal	1205-17-0	16.4	0.85	[17, 18]
Cyclamen aldehyde	103-95-7	22	1.64	[19]
Methoxyhydratropaldehyde (4-Methoxy-α-methyl benzenpropanal)	5462-06-6	23.6	1.32	[17]

**Table 4.** Fragrances for which only single-center clinical data are available or for which “R43”-labeling (“none*”) plus a sensitization potency according to SAR analysis is possible (“+”) or probable (“++”).

Substance (INCI name or name of perfume according to CosIng)	CAS number	Clinical data	SAR
Ambrettolide	7779-50-2	limited	+
Carvacrol	499-75-2	limited	+
Citrus paradisi	8016-20-4	none*	n.a.
Cuminaldehyde	122-03-2	limited	+
Cyclopentadecanone	502-72-7	limited	+
trans-trans-delta-Damascone	71048-82-3	limited	+
2,4-Dimethyl-3-cyclohexen-1- carboxaldehyde	68039-49-6	none*	+
Dimethyltetrahydro benzaldehyde	68737-61-1	limited	+
Ethyl vanillin	121-32-4	limited	+
Heliotropine	120-57-0	limited	+
Isoamyl salicylate	87-20-7	limited	++
Isolongifoleneketone	33407-62-4	limited	+
Longifolene	475-20-7	none*	+
Mentha arvensis	68917-18-0	none*	n.a.
Methoxycitronellal	3613-30-7	limited	+
Methyl cinnamate	103-26-4	limited	++
Methylionantheme	55599-63-8	limited	+
5-Methyl-alpha-ionone	79-69-6	limited	+
Myrcene	123-35-3	limited	++
Myrtenol	515-00-4	limited	+
Nerol	106-25-2	limited	++
Nerolidol (isomer nonspecified)	7212-44-4	limited	++
Nopyl acetate	128-51-8	limited	+
Phytol	150-86-7	limited	+
Rhodinol	6812-78-8	limited	+
trans-Rose ketone-5	39872-57-6	limited	++
